# Mitochondrial DNA depletion induces AD like phenotypes in neuronal and glial cells

**DOI:** 10.1002/alz.089073

**Published:** 2025-01-03

**Authors:** Carmen Romero Molina, Colton R Lysaker, Anysja Roberts, Caleb Gilmore, Wen Yi See, Yiyuan Liu, Judy Pa, Russell H Swerdlow, Alison M. Goate, Shea J Andrews, Heather M Wilkins

**Affiliations:** ^1^ Icahn School of Medicine at Mount Sinai, New York City, NY USA; ^2^ Ronald M. Loeb Center for Alzheimer’s disease, New York City, NY USA; ^3^ University of Kansas Medical Center, Kansas City, KS USA; ^4^ Icahn School of Medicine at Mount Sinai, New York, NY USA; ^5^ Ronald M. Loeb Center for Alzheimer’s disease, New York, NY USA; ^6^ Alzheimer’s Disease Cooperative Study (ADCS), University of California, San Diego, La Jolla, CA USA; ^7^ Alzheimer’s Disease Research Center, Keck School of Medicine, University of Southern California, Los Angeles, CA USA; ^8^ University of Southern California, Los Angeles, CA USA; ^9^ Neurosciences Graduate Program, University of California, San Diego, La Jolla, CA USA; ^10^ UC San Diego Health, La Jolla, CA USA; ^11^ Stevens Neuroimaging and Informatics Institute, University of Southern California, Los Angeles, CA USA; ^12^ University of Kansas Alzheimer’s Disease Research Center, Fairway, KS USA; ^13^ Ronald M. Loeb Center for Alzheimer’s Disease, Friedman Brain Institute, Icahn School of Medicine at Mount Sinai, New York, NY USA; ^14^ Department of Genetics and Genomic Sciences, New York, NY USA; ^15^ Department of Genetics and Genomic Sciences, Icahn School of Medicine at Mount Sinai, New York, NY USA; ^16^ MSSM, New York, CA USA; ^17^ Ronald M. Loeb Center for Alzheimer’s Disease, Dept. of Neuroscience, Icahn School of Medicine at Mount Sinai, New York, NY USA; ^18^ Australian National University, Canberra, CA USA; ^19^ University of California, San Francisco, San Francisco, CA USA

## Abstract

**Background:**

Mitochondrial dysfunction is an early and prominent feature of Alzheimer’s disease (AD). We have recently published that lower brain mitochondrial DNA copy number (mtDNAcn) is associated with increased risk of AD neuropathological change and reduced cognitive performance. Here, we addressed how mtDNAcn affects cell‐type specific phenotypes.

**Method:**

iPSC derived neural progenitor cells were infected with a lentivirus encoding a mitochondrial targeted exonuclease under the control of a Tet promoter. mtDNA was depleted with doxycycline and cells were differentiated into neurons or astrocytes using established protocols (StemCell Technologies). iPSC derived astrocyte migration/chemotaxis was measured in the presence of ATP. Microglial BV2 cells were treated with ethidium bromide (etbr) to reduce mtDNAcn. Cells were characterized by RNAseq, ELISAs, Western Blotting, and Seahorse experiments. BV2 cells were challenged with latex beads and zymosan‐pHrodo to analyze phagocytosis. BV2 cells were stimulated with LPS to measure cytokine release. After BV2 stimulation, fresh media was conditioned for 6 hours and collected to treat neurons (SH‐SY5Y) and human primary astrocytes.

**Result:**

mtDNAcn reduction led to reduced oxidative phosphorylation and a mild reduction in proliferation for glial cell types. iPSC derived neurons and astrocytes with mtDNAcn reduction showed increased expression of STING and reduced oxidative phosphorylation. iPSC derived neurons with mtDNAcn reduction showed increased Aβ secretion and changes to tau homeostasis. iPSC derived astrocytes with mtDNAcn reductions had reduced migration/chemotaxis but increased TNFα production. RNAseq data from BV2 cells showed an increase in the hypoxia signature and cellular senescence, coupled with a reduction in biosynthetic process and response to interferon. No changes were observed in phagocytic capacity. However, after LPS stimulation, etbr‐treated cells showed a reduction in the production of IL1B and IL6 cytokines, suggesting an impairment in the response to challenge. Neurons exposed to media from etbr‐treated BV2 cells showed an increase in MAPT and APOE expression.

**Conclusion:**

mtDNAcn reduction can lead to AD pathological hallmarks including changes in Aβ secretion, tau homeostasis, and glial cell phenotypes. mtDNAcn reduction impairs mitochondrial respiration in glial cells, which affects the production of cytokines and leads to cell senescence. Cytokines released by glial cells with reduced mtDNAcn affected neuronal expression of AD‐related genes.

